# Research on antibiotic resistance in *Helicobacter pylori*: a bibliometric analysis of the past decade

**DOI:** 10.3389/fmicb.2023.1208157

**Published:** 2023-06-14

**Authors:** Chengzhi Yuan, Chang Yu, Qifang Sun, Meng Xiong, Sainan Zhou, Meiyan Zeng, Houpan Song

**Affiliations:** ^1^Hunan Provincial Key Laboratory of Traditional Chinese Medicine Diagnostics, Hunan University of Chinese Medicine, Changsha, Hunan, China; ^2^School of Medicine, Hunan University of Chinese Medicine, Changsha, Hunan, China; ^3^School of Traditional Chinese Medicine, Hunan University of Chinese Medicine, Changsha, Hunan, China; ^4^The First Affiliated Hospital of Hunan University of Chinese Medicine, Changsha, Hunan, China

**Keywords:** *Helicobacter pylori*, antibiotic resistance, microbiome, bibliometrics, visualization

## Abstract

Resistance of *Helicobacter pylori* (*H. pylori*) to antibiotics has reached alarming levels worldwide, and the efficacy of the *H. pylori* eradication treatment has decreased dramatically because of antibiotic resistance. To gain a more comprehensive understanding of the development status, research hotspots, and future trends related to *H. pylori* antibiotic resistance, we conducted a thorough retrospective analysis via the bibliometrics method. We searched the Science Citation Index Expanded of the Web of Science Core Collection for all pertinent articles on *H. pylori* antibiotic resistance from 2013 to 2022. R-bibliometrix, CiteSpace, and VOSviewer tools were utilized to depict statistical evaluations in order to provide an unbiased presentation and forecasts in the field. We incorporated a total of 3,509 articles related to *H. pylori* antibiotic resistance. Publications were inconsistent prior to 2017, but steadily increased after 2017. China generated the most papers and the United States of America received the most citations and the highest H-index. *Baylor College of Medicine* was the most influential institution in this field, with the highest number of publications and citations, as well as the highest H-index. *Helicobacter* was the most productive journal, followed by the *World Journal of Gastroenterology* and *Frontiers in Microbiology*. T*he World Journal of Gastroenterology* had the highest citation. Graham, David Y was the most productive and cited author. Clarithromycin resistance, prevalence, gastric cancer, quadruple therapy, sequential therapy, 23S rRNA, whole genome sequencing, bismuth, and probiotics appeared with a high frequency in the keywords. The top keywords with the highest citation bursts were vonoprazan, RdxA, biofilm formation, and fatty acid chain. Our research illustrated a multi-dimensional facet and a holistic knowledge structure for *H. pylori* antibiotic resistance research over the past decade, which can serve as a guide for the *H. pylori* research community to conduct in-depth investigations in the future.

## 1. Introduction

The gram-negative spiral bacterium *Helicobacter pylori* (*H. pylori*) colonizes over 50% of all humans because the immune system fails to eliminate it from the upper gastrointestinal tract regularly (Fukase et al., 2008). As the main cause of gastritis, gastric ulcer, duodenal ulcer, and gastric cancer, *H. pylori* infection causes worldwide concern (Sugano et al., 2015). The third most common cause of cancer death worldwide is gastric cancer, which is mainly caused by infection with *H. pylori* (Chiang et al., 2021).

Traditionally, *H. pylori* had been treated with a standard triple therapy (STT) consisting of proton pump inhibitors (PPIs), clarithromycins (CAMs), and amoxicillins (AMPCs) (Malfertheiner et al., 2022). However, *H. pylori* eradication rates have declined to unacceptable levels because of high resistance to metronidazole and clarithromycin with the widespread use STT regimen (Ho et al., 2022). According to certain research, eliminating *H. pylori* with STT did not even reach 50% (Dore et al., 2000; Luther et al., 2010; Venerito et al., 2013). Accordingly, in locations with high clarithromycin resistance levels (>15%), current international standards suggest bismuth quadruple therapy (PPI, tetracycline, bismuth, and metronidazole) or concomitant non-bismuth quadruple therapy (PPI, amoxicillin, clarithromycin, and metronidazole) for 14 days (Fallone et al., 2016; Chey et al., 2017; Malfertheiner et al., 2022). Due to the high antibiotic resistance, *H. pylori* is included in the WHO Global Priority List of Antibiotic-Resistant Bacteria (Tacconelli et al., 2018), underscoring the urgency of developing an eradication drug that targets and kills the bacteria. Alternatives (such as quadruple regimens, sequential regimens, concomitant regimens, and levofloxacin-containing triple regimens) differ greatly in effectiveness (Nyssen et al., 2016; Thung et al., 2016).

In recent years, as research on *H. pylori* progresses, there has been a significant increase in the volume of scholarly literature produced in this field. Faced with the vast body of publications, traditional analysis methods are no longer sufficient to accurately capture the evolving developments of this research area. Bibliometric analysis is an approach that statistically and quantitatively examines academic publications (Kokol et al., 2021). By utilizing literature retrieval system and metrology to analyze publications, researchers can gain a comprehensive understanding of publications from multiple perspectives. This enables them to evaluate the history, current state, and future potential of these publications, as well as their quantity and quality. As a result of bibliometric analysis, network knowledge maps can be drawn, new trends can be predicted, and recent advancements in a specific field can be displayed (Guler et al., 2016; Sainan et al., 2022). The current utilization of bibliometrics in the medical field can provide valuable insights into academic trends, pharmacotherapy, and disease progression, as well as the overall trends in healthcare (Thompson and Walker, 2015; Kokol et al., 2021). A number of bibliometric assessments have been published in the biological sector in the past few years, but there are few bibliometric analyses available on the antibiotic resistance in *H. pylori*. Consequently, this research employs two bibliometric applications, VOSviewer and CiteSpace, to measure the extent of research on *H. pylori* antibiotic resistance and recognize the most popular scientific questions in recent years. It is hoped that the results will provide clinicians and related researchers with useful information and further assist them in conducting more in-depth investigations.

## 2. Materials and methods

### 2.1. Search and inclusion criteria

**Figure 1:**
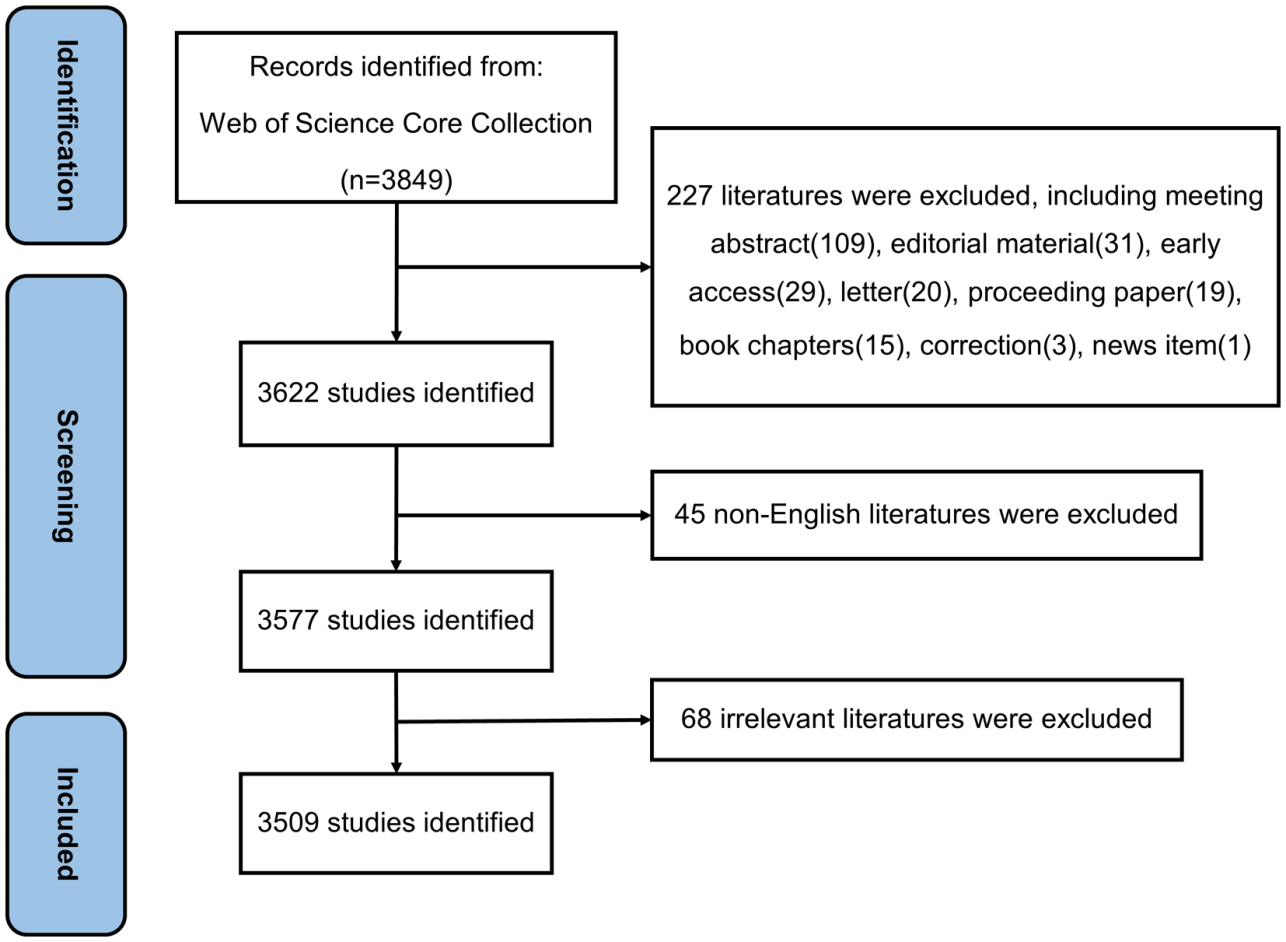
Flowchart of screening process.

The data were sourced from the Science Citation Index Expanded (SCI-E) within the Web of Science Core Collection (WoSCC). The search strategies were shown as follows: TS = (*Helicobacter pylori* or HP or *H. pylori*) AND TS = (drug resistance or drug tolerance or antibiotic resistance or antimicrobial resistance or antimicrobial agent or antibiotic). Here, TS referred to the subject matter as denoted by WoSCC. The time span of this research was: 2013–2022. To ensure data accuracy, the literature production search was completed on a single day (11 January 2023), and only articles and reviews written in English were considered. During the search, 3,509 publications were found. A detailed description of the screening process can be found in Figure 1. For further analysis, relevant articles were exported and stored in text file (including the entire record and cited references).

### 2.2. Data processing

We extracted the raw data from WoSCC, which primarily consisted of year of publication, author, country/region, institution, journal, H-index, references, and keywords. Initially, we eliminated duplicate authors and misspelled elements manually. Secondly, we merged names of the same country represented differently. Subsequently, we utilized a synonym database file to combine some duplicate words into a single word, corrected misspelled elements, and removed irrelevant words before analyzing the data using CiteSpace and VOSviewer.

### 2.3. Data analysis

#### 2.3.1. Data analysis based on bioinformatics

As an online platform, bioinformatics^1^ was used to generate the following images: the world map of publication distribution, the heat map of top 10 countries in publication rankings, the pie chart of the top 10 research areas, and the bubble chart of the top 10 most cited papers. It is important to note that the bioinformatics platform integrates and utilizes the capabilities of R-bibliometrix, a comprehensive R package for bibliometric analysis.

#### 2.3.2. Data analysis based on CiteSpace

Developed by Professor Chen, CiteSpace is a Java application for bibliometric analysis (Chen, 2004). In CiteSpace, the dual-map overlay, timeline view, and citation bursts of keywords and references are all used to examine knowledge fields and trends visually (Liu et al., 2021). The CiteSpace tool can be used to identify emerging topics based on references with strong citation burstness. We imported the retrieved literature into CiteSpace for deduplication, and then analyzed the processed results. In our study, the CiteSpace settings were as follows: time slicing was performed from 2013 to 2022, with 1-year intervals per slice. Text proceedings were set as default. The pathfinder and pruning sliced networks were used to prune unnecessary links. The “Overlay Map” button was used to generate the dual-map overlay, the “Layout” button to create the timeline view of keywords and references, and the “Burstness” button to display the citation bursts of keywords and references.

#### 2.3.3. Data analysis based on VOSviewer

Developed by Leiden University’s Center for Science and Technology Research, VOSviewer is a network analysis software for scientific metrics. With VOSviewer, closely related nodes can be clustered by color, with the same color indicating greater correlations (van Eck and Waltman, 2010). Nodes’ sizes reflect the number of publications, and lines’ thicknesses reflect the strength of their relationships. We used VOSviewer to classify references and keywords with high co-occurrence frequencies into clusters and create a density visualization of keywords.

## 3. Results

### 3.1. Annual quantitative distribution of publications

**Figure 2:**
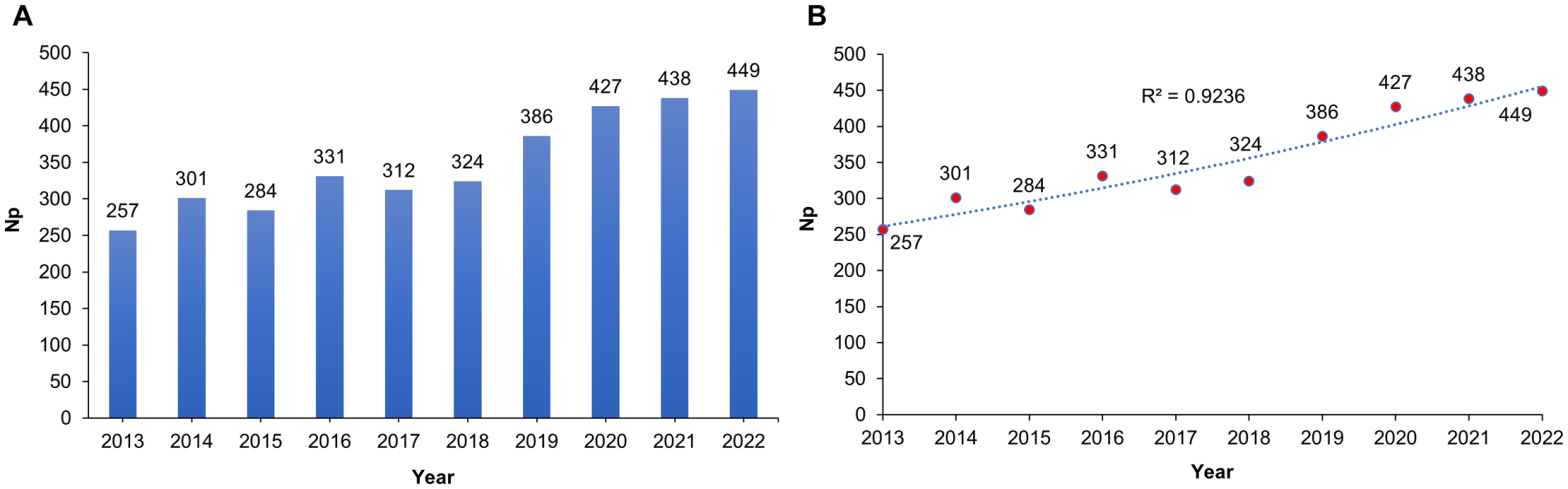
**(A)** The number of annual publications on *Helicobacter pylori* antibiotic resistance research between 2013 and 2022. The vertical axis indicates the quantity of publications while the horizontal axis corresponds with the publication year. **(B)** Curve-fitting analysis of the overall annual growth trend for publications.

A graph showing annual trends in publications related to antibiotic resistance in *H. pylori* is shown in Figure 2A. There are 3,509 publications on antibiotic resistance in *H. pylori*, of which 2,682 are regular articles and 827 are review articles. A fluctuating upward trend was observed in the number of publications (Np) concerning antibiotic resistance in *H. pylori* between 2013 and 2022. A peak in the number of yearly papers occurred in 2022, when the Np reached 449 compared to 257 in 2013. Figure 2B displays a polynomial-fitting curve that illustrates the annual trend in paper publishing. Despite some minor fluctuations over the last decade, there has been a general inclination toward an increase in the number of published articles, as indicated by a correlation coefficient of 0.92. In general, these data suggest that *H. pylori* antibiotic resistance research has been of major interest and progressed rapidly in recent years.

### 3.2. Country and regional distribution

**Figure 3:**
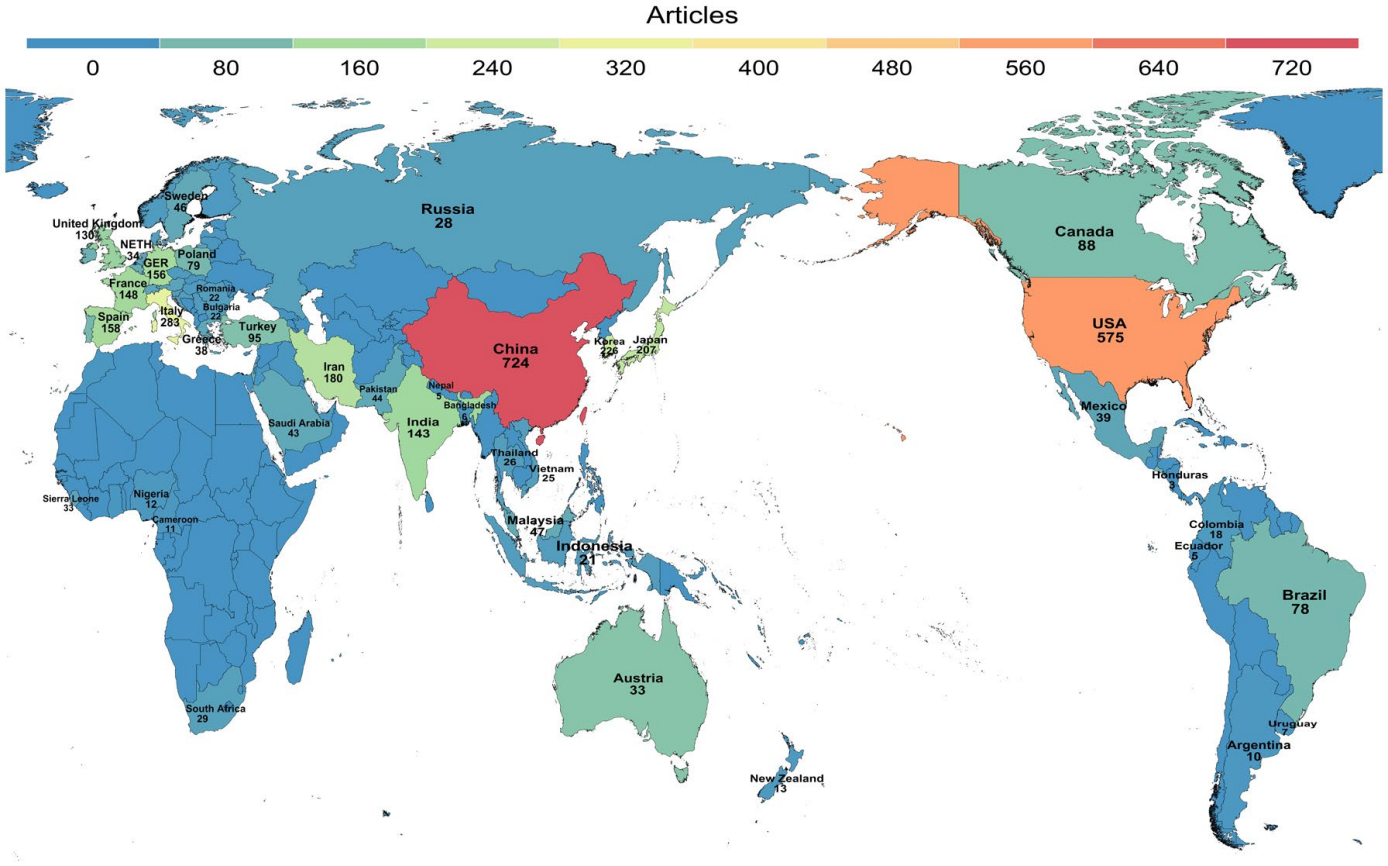
The contribution of every country based on publication counts is depicted on the world map.

**Figure 4:**
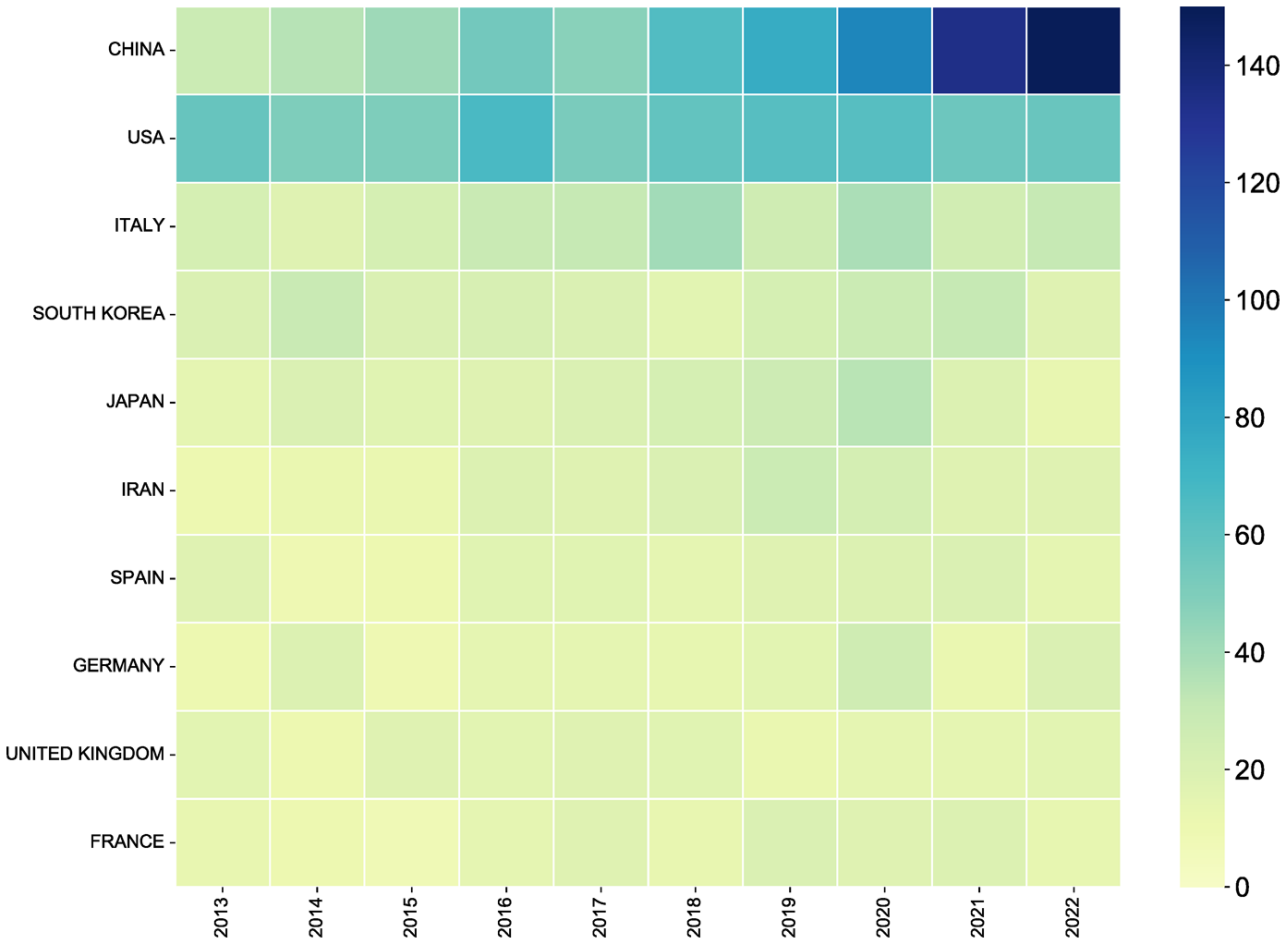
Top 10 countries in terms of annual publications on *Helicobacter pylori* antibiotic resistance research, from 2013 to 2022. The square’s colors represent the number of papers. As the color changes from light green to dark blue, the number of articles issued increases.

There were 123 countries/regions contributing to the publication of research on antibiotic resistance in *H. pylori* between 2013 and 2022. An overview of the geographic distribution of papers on antibiotic resistance in *H. pylori* among all countries and regions can be found in Figure 3. Table 1 shows the top 10 countries and regions based on Np. R-bibliometrix software was employed to analyze all data, resulting in the automatic merging of England, Wales, and Scotland data for the country analysis, which was eventually presented as the United Kingdom. As shown in Table 1, China had the highest number of published papers (724/20.63%), followed by the United States (575/16.39%) and Italy (283/8.07%). The top three countries by number of citations were the United States (21,215), China (11,112), and Italy (10,475). Additionally, the H-index rankings in descending order were the United States (72), China (55), and Italy (46). Although Np was relatively low in the United Kingdom, France, and Germany, the number of average citations (Na) was significantly higher in these countries. It may be related to the generally higher quality of papers produced in United Kingdom, France, and Germany. The annual paper publication of the top 10 countries/regions is illustrated in Figure 4.

**Table 1:** Top 10 most productive countries/regions.

Rank	Country/region	(NP)	% of (3,509)	(Nc)	(Na)	H-index
1	China	724	20.63%	11,112	15.35	55
2	United States	575	16.39%	21,215	36.90	72
3	Italy	283	8.07%	10,475	37.01	46
4	South Korea	226	6.44%	3,425	15.15	31
5	Japan	207	5.90%	3,415	16.50	33
6	Iran	180	5.13%	2,835	15.75	29
7	Spain	158	4.50%	4,788	30.30	36
8	Germany	156	4.45%	6,913	44.31	34
9	United Kingdom	154	4.39%	8,498	55.18	39
10	France	148	4.22%	6,239	42.16	32

### 3.3. Most productive institutions

A list of the top 10 institutions associated with the highest number of publications related to *H. pylori*’s antibiotic resistance was presented in Table 2. In terms of Np, Baylor College of Medicine ranked first (78). Shanghai Jiao Tong University was next (57), and Seoul National University was third (53). Baylor College of Medicine also had the highest Nc (3,653) and H-index (34) among all institutions. Baylor College of Medicine is a private medical school and is considered one of the most distinguished medical schools in America. The university has also made remarkable contributions to the domain of biomedicine.

**Table 2:** Top 10 institutions with the highest number of publications in *Helicobacter pylori* antibiotic resistance research.

Rank	Institution	Country/region	Np	Nc	H-index
1	Baylor College of Medicine	United States	78	3,653	34
2	Shanghai Jiao Tong University	China	57	1,792	24
3	Seoul National University	South Korea	53	912	18
4	Nanchang University	China	51	960	18
5	University of Bologna	Italy	44	1,177	20
6	National Taiwan University	Taiwan	43	2,134	21
7	Michael E. DeBakey VA Medical Center	United States	41	1,059	16
8	Peking University	China	40	968	16
9	Kaohsiung Medical University	Taiwan	38	1,330	21
10	Sun Yat-sen University	China	37	989	16

### 3.4. Most influential authors

A total of 16,873 authors have contributed to the development of antibiotic resistance associated with *H. pylori*. Table 3 presents the top 10 most productive authors and the most cited authors. Among the most productive team/labs, Graham, David Y’s team/lab published 44 articles, followed by Gisbert, Javier P (39 articles), and Megraud, Francis (31 articles). As to citations in this field, Graham, David Y topped the list with 2,807 local citations, followed by Wu, Ming-Shiang (1,579 local citations), and Gisbert, Javier P (1,544 local citations), while the rest of the authors had less than 1,500 local citations. Especially noteworthy, there is no doubt that Graham, David Y. is the most active researcher and has significantly contributed to the growth of the subject as evidenced by his largest number of publications and citations.

**Table 3:** Top 10 most productive and cited authors in *Helicobacter pylori* antibiotic resistance research.

Rank	Highly published authors	Country	Np	Highly cited author	Country	Nc
1	Graham, David Y	United States	44	Graham, David Y	United States	2,807
2	Gisbert, Javier P	Spain	39	Wu, Ming-Shiang	China	1,579
3	Megraud, Francis	France	31	Gisbert, Javier P	Spain	1,544
4	Yamaoka, Yoshio	Indonesia	28	Lu, Hong	China	1,261
5	Lu, Hong	China	27	Lee, Yi-Chia	China	1,199
6	Kim, Nayoung	South Korea	27	Moss, Steven F	United States	1,104
7	Wu, Deng-Chyang	China	26	Supuran, Claudiu T	Italy	1,018
8	Wu, Ming-Shiang	China	25	Liou, Jyh-Ming	China	937
9	Liou, Jyh-Ming	China	24	Megraud, Francis	France	806
10	Supuran, Claudiu T	Italy	21	Lin, Jaw-Town	China	800

### 3.5. Distribution of academic journals and disciplines

During the period 2003–2013, 3,509 articles were published in 1,038 journals. The top 10 journals with the most publications are included in Table 4 along with their most recent impact factors (IF). With 220 publications, *Helicobacter* accounted for 6.255% of all articles, followed by *World Journal of Gastroenterology* (127, 3.611%), and *Frontiers in Microbiology* (71, 2.019%). Among the top 10 journals, three belonged to the Q1 JCR division, while five had an IF exceeding five. A journal’s citations can be examined to identify its core journals, the top 10 most frequently cited journals are shown in Table 4. Among cited journals, *World Journal of Gastroenterology* was the most frequently cited (3,930), followed by *Helicobacter* (3,906), and *Gastroenterology* (1,983). In the Q1 Journal Citation Reports (JCR) for 2020, seven journals were at the division, and four out of the top 10 most cited journals had an IF exceeding 10.

**Table 4:** The top 10 journals and cited journals of *Helicobacter pylori* antibiotic resistance research.

Rank	Top journals	Np	2021 IF	2021 JCR	Cited journals	Nc	2021 IF	2021 JCR
1	Helicobacter	220	5.182	Q2	World Journal of Gastroenterology	3,930	5.374	Q2
2	World Journal of Gastroenterology	127	5.374	Q2	Helicobacter	3,906	5.182	Q2
3	Frontiers in Microbiology	71	6.064	Q1	Gastroenterology	1,983	33.883	Q1
4	PLOS One	64	3.752	Q2	Frontiers in Microbiology	1,829	6.064	Q1
5	Antibiotics Basel	49	5.222	Q1	Gut	1,629	31.795	Q1
6	Molecules	36	4.927	Q2	PLOS One	1,541	3.752	Q2
7	Infection and Drug Resistance	35	4.177	Q2	Alimentary Pharmacology and Therapeutics	1,249	9.524	Q1
8	Journal of Gastroenterology and Hepatology	35	4.369	Q2	American Journal of Gastroenterology	1,097	12.045	Q1
9	International Journal of Molecular Sciences	34	6.208	Q1	Cochrane Database of Systematic Reviews	864	11.874	Q1
10	Scientific Reports	32	4.997	Q2	International Journal of Molecular Sciences	850	6.208	Q1

**Figure 5:**
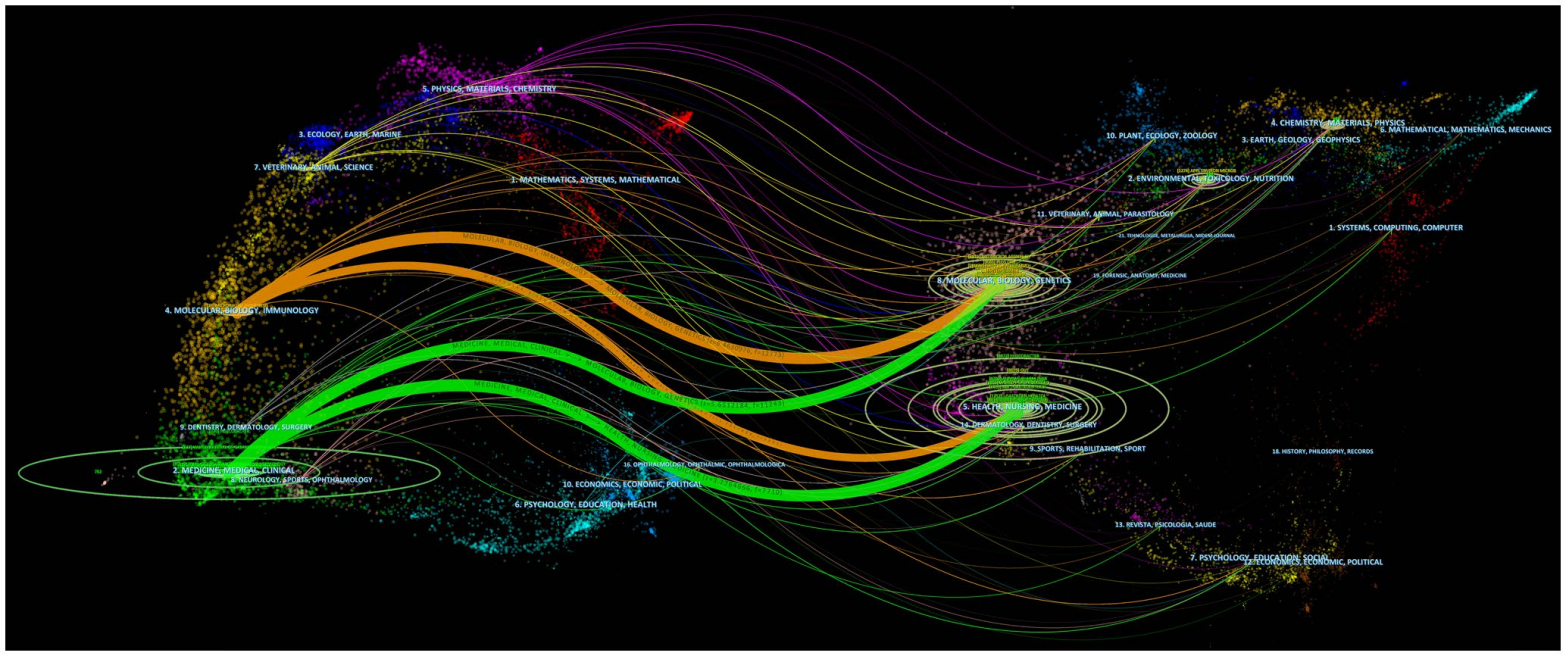
A biplot overlay of journals on *Helicobacter pylori* antibiotic resistance research. Every point depicted on the map corresponds to a journal, and the map is partitioned into two sections, featuring the citing map on the left side and the cited map on the right side. The curve is the citation line. The ellipse in the left panel denotes the quantity of publications associated with a journal and displays the proportion of authors to the number of publications; The length of the ellipse indicates the number of authors and the width of the ellipse indicates the number of publications.

**Figure 6:**
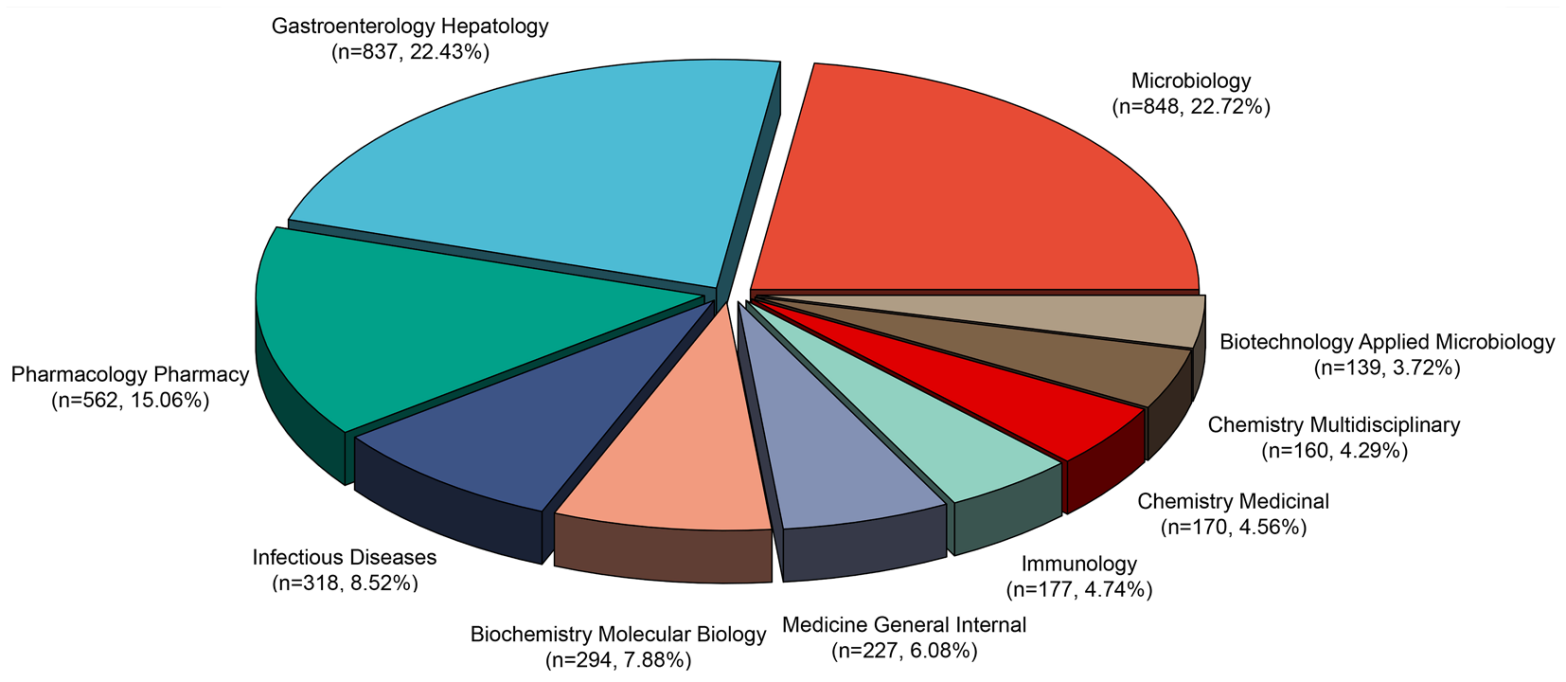
Top 10 research fields with the most published articles on antibiotic resistance in *Helicobacter pylori*.

Chen and Leydesdorff developed a dual-map overlay analysis to reveal patterns within a scientific portfolio that match a global scientific literature map (Chen and Leydesdorff, 2014). As shown in Figure 5, a dual-map overlay depicts the publication of antibiotic resistance studies in *H. pylori* between 2013 and 2022. In this visual description, the retrieved records’ publication locations are represented by the left clusters, while the right clusters represent the locations where they were cited. The label is a representation of the topic addressed by the journal. The colored curves in the visualization correspond to lines of references, which originate from the citing map and extend toward the cited map. and this interaction illustrates the connections between different fields of research. The citation paths in our dataset were divided into four main categories. There were two main domains that covered most of the records: (1) medicine, medical, clinical, and (2) molecular, biology, immunology. Research content was primarily influenced by the following domains: (1) molecular, biology, genetics, and (2) health, nursing, and medicine. In total, the identified publications were categorized into 121 research areas. According to Figure 6, Microbiology was the most represented research area (848 records, 24.10% of all articles), followed by Gastroenterology Hepatology (837, 23.79% of all articles), and Pharmacology Pharmacy (562, 15.97% of all articles).

### 3.6. Research hotspots and frontier analysis

#### 3.6.1. Most cited articles

**Figure 7:**
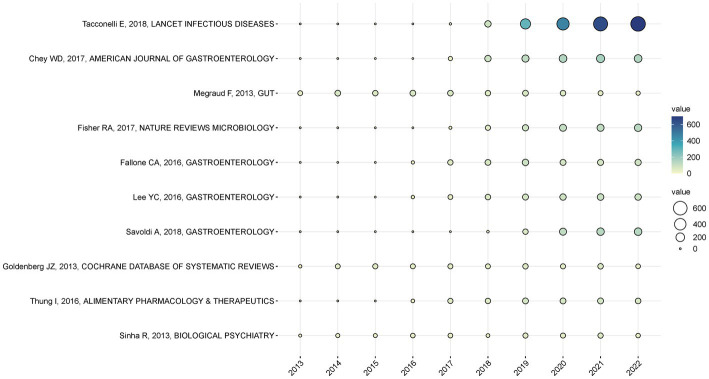
Annual citation of the top 10 most cited papers globally. Citations to the article are represented by circle’s size and color. Circles with a larger size and colors ranging from light green to dark blue indicate more times an article has been cited and its influence.

In bibliometric analysis, citation frequency is an important and representative indicator that measures academic influence and community display (Bruer, 1982; Nieminen et al., 2006; Poomkottayil et al., 2011). A ranking of the top 10 most cited articles is shown in Table 5. They were all published between 2013 and 2022, and all of them attracted more than 300 citations. With 2,212 citations, “Discovery, research, and development of new antibiotics: the WHO priority list of antibiotic-resistant bacteria and tuberculosis” published in 2018 is the most cited article in this field. In the article, antimicrobial-resistant bacteria are prioritized using multicriteria decision analysis to determine the most important antibiotic-resistant bacteria to eradicate. Figure 7 illustrates the annual count of worldwide citations for the top 10 most frequently cited papers.

**Table 5:** Top 10 cited articles of *Helicobacter pylori* antibiotic resistance research.

Rank	PMID number	Journal	First author	Total citation	Average citation
1	PMID: 29276051	Lancet Infectious Diseases	Tacconelli E	2,212	368.67
2	PMID: 28071659	American Journal of Gastroenterology	Chey WD	692	98.86
3	PMID: 22580412	Gut	Megraud F	541	49.18
4	PMID: 28529326	Nature Reviews Microbiology	Fisher RA	493	70.43
5	PMID: 27102658	Gastroenterology	Fallone CA	460	57.5
6	PMID: 26836587	Gastroenterology	Lee YC	456	57
7	PMID: 29990487	Gastroenterology	Savoldi A	448	74.67
8	PMID: 23728658	Cochrane Database of Systematic Reviews	Goldenberg JZ	417	37.91
9	PMID: 26694080	Alimentary Pharmacology and Therapeutics	Thung I	396	49.5
10	PMID: 23541000	Biological Psychiatry	Sinha R	335	30.45

#### 3.6.2. Co-cite reference analysis

**Figure 8:**
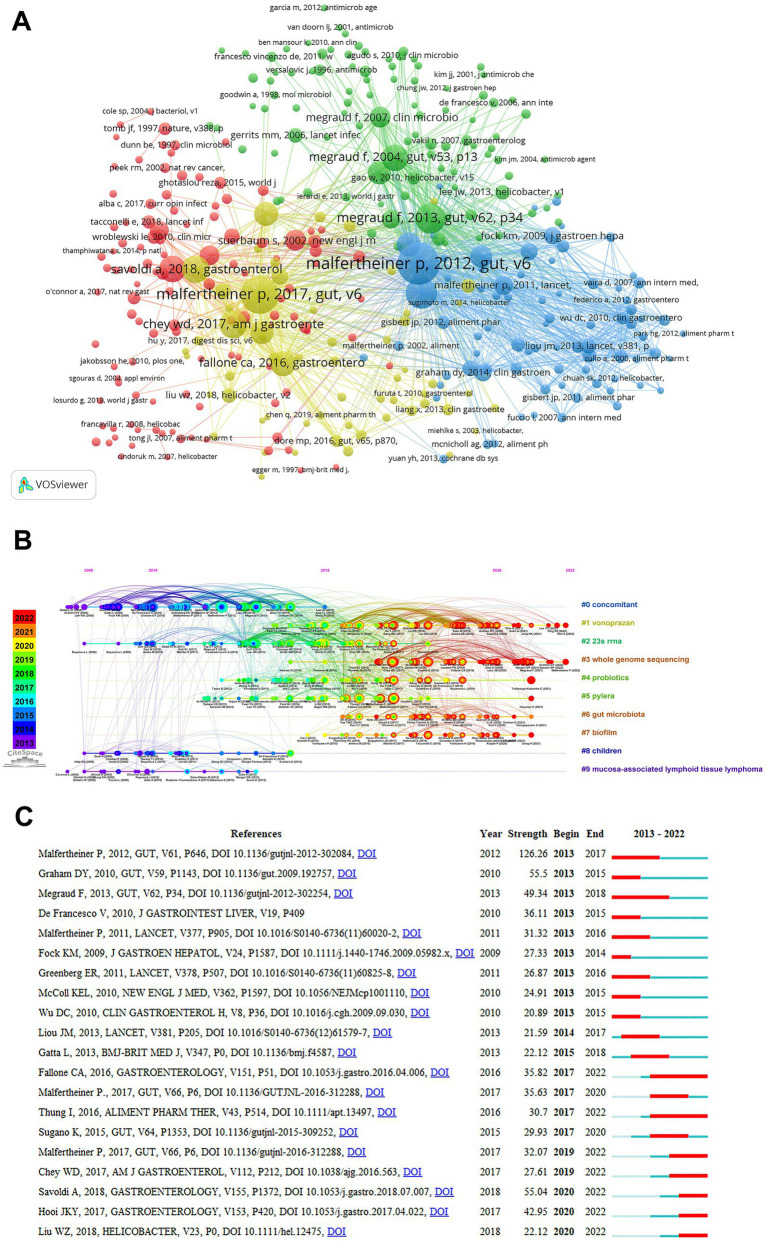
Mapping based on co-cited references from *Helicobacter pylori* antibiotic resistance research. **(A)** Network diagram of co-citation analysis of references cited more than 30 times. **(B)** The timeline view of co-cited references. **(C)** The top 20 references with the highest citation bursts. References were most influential between begin and end. The blue bar indicates that the reference has been published, while the red bar indicates that the citations have burst.

“Co-cited references” are references that are cited by multiple publications simultaneously and can be used to represent the knowledge base of a particular area (Wang et al., 2022). Considering the massive amount of references cited, the incorporated document had to contain at least 30 citations. A total of 353 references were selected for co-citation analysis out of 12,4,113 publications mentioned in the retrieved documents. Table 6 lists the top 10 co-cited references. Malfertheiner P’s “Management of *Helicobacter pylori* infection-the Maastricht IV/ Florence Consensus Report”(593) and “Management of *Helicobacter pylori* infection-the Maastricht V/Florence Consensus Report”(506) published in GUT were the most co-cited references in relation to antibiotic resistance in *H. pylori* (Malfertheiner et al., 2012, 2017). Co-cited references are visualized as a network in Figure 8A. Nodes’ sizes indicate how often co-cited references occur, and the distance between nodes indicates how strong their association is. Co-cited references were subsequently grouped according to their indexing terms. A timeline view of distinct clusters can be seen in Figure 8B. The top 1 clusters of co-cited references consisted of concomitant, vonoprazan, 23 s rRNA, whole genome sequencing, probiotics, pylera, gut microbiota, biofilm, children, and mucosa-associated lymphold tissue lymphoma. In Figure 8C, we present the top 20 references with the highest citation bursts. Among the studies, Malfertheiner P’s study has the highest strength (126.26). Throughout his article, he outlines best practices for treating *H. pylori* infections in a variety of clinical scenarios based on the best available evidence. Furthermore, several articles are still experiencing citation bursts, such as “Prevalence of Antibiotic Resistance in *Helicobacter pylori*: A Systematic Review and Meta-analysis in World Health Organization Regions” suggesting that these topics are expected to remain popular for a while and may become a potential area of focus in research on antibiotic resistance in *H. pylori*.

**Table 6:** Top 10 co-cited references for *Helicobacter pylori* antibiotic resistance research.

Rank	PMID number	Journal	First author	Year	Citation
1	PMID: 22491499	Gut	Malfertheiner P	2012	593
2	PMID: 27707777	Gut	Malfertheiner P	2017	506
3	PMID: 20525969	Gut	Graham DY	2010	369
4	PMID: 22580412	Gut	Megraud F	2013	354
5	PMID: 28071659	American Journal of Gastroenterology	Chey WD	2017	300
6	PMID: 29990487	Gastroenterology	Savoldi A	2018	290
7	PMID: 15306603	Gut	Megraud F	2004	285
8	PMID: 28456631	Gastroenterology	Hooi JKY	2017	279
9	PMID: 27102658	Gastroenterology	Fallone CA	2016	259
10	PMID: 26694080	Alimentary Pharmacology and Therapeutics	Thung I	2016	245

#### 3.6.3. Keywords analysis

**Figure 9:**
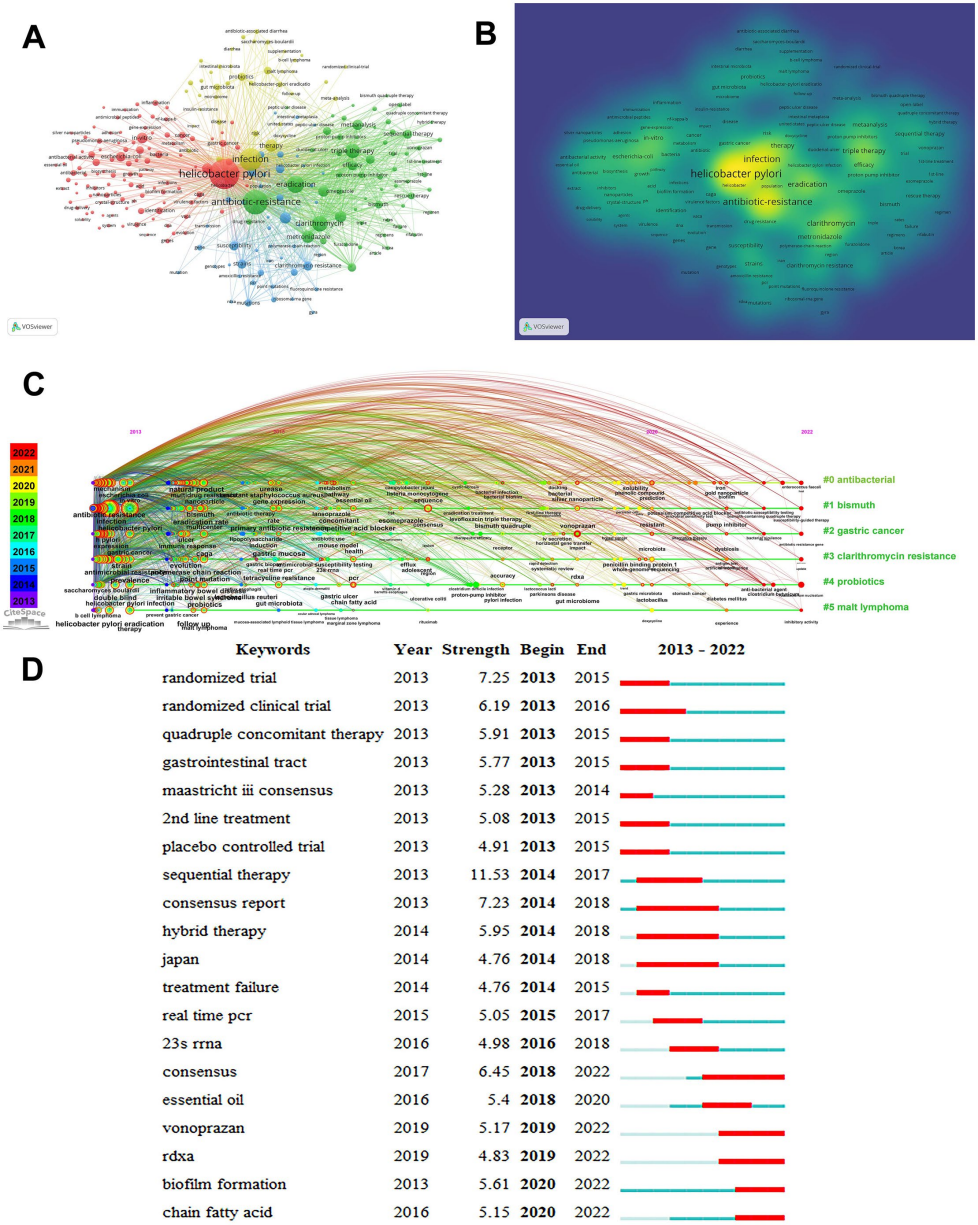
Mapping based on keywords from *Helicobacter pylori* antibiotic resistance research. **(A)** The co-occurrence network of frequently occurring keywords in *H. pylori* antibiotic resistance research. **(B)** Hot map of keywords research. More intense colors indicate higher keyword frequency. **(C)** The timeline view of keywords. **(D)** The top 20 most frequently cited keywords with the highest citation bursts.

The keyword co-occurrence enables us to identify hot spots and directions in this field of research. With VOSviewer, we extracted 260 keywords (the minimum number of occurrences was 20) and merged words with similar meanings. Based on the results obtained in Figure 9A, four clusters with different colors are shown, corresponding to four different research directions and research scopes. Cluster 1 (red) contains 112 keywords, including gastric cancer, inflammation, mechanisms, identification, biofilm formation, crystal structure, *Escherichia coli*, and virulence factors. Cluster 2 (green) contains 63 keywords, including triple therapy, sequential therapy, eradication, clarithromycin, metronidazole, bismuth, proton pump inhibitor, meta-analysis, and levofloxacin. Cluster 3 (blue) contains 46 keywords, including antimicrobial susceptibility, PCR, point mutations, genotypes, prevalence, clarithromycin resistance, metronidazole resistance, 23S ribosomal RNA, 13C-urea breath test. Cluster 4 (yellow) contains 39 keywords, including probiotics, gut microbiota, dysbiosis, inflammatory bowel disease, antibiotic associated diarrhea, *Saccharomyces boulardii*, chain fatty acids, prevention, malt lymphoma. These keywords represent the hotspots for research related to *H. pylori* resistance. We employed a blend of qualitative and quantitative approaches, as shown in Supplementary Table 1, to analyze the clustering of keyword networks, which allowed us to pinpoint significant themes, hotspots, and emerging trends (Kokol et al., 2022). Keyword density maps (Figure 9B) can intuitively display these high-frequency keywords. Figure 9C shows the dynamic evolution of keywords clusters over time. In total, 6 clusters have been identified, including antibacterial, bismuth, gastric cancer, clarithromycin resistance, probiotics, and malt lymphoma. Figure 9D shows the top 20 keywords with the strongest bursts. In terms of keyword intensity, sequential therapy (strength = 11.53) was the top keyword, followed by randomized trial (7.25) and consensus report (7.23). It is noteworthy that the keywords vonoprazan, RdxA, biofilm formation, and chain fatty acid have been heavily cited recently, which indicates the future hotspots for research on *H. pylori*.

## 4. Discussion

### 4.1. General information

The WHO designated eradication failure of *H. pylori* as an immediate priority in 2017. Similar to other efforts to improve *H. pylori* eradication rates, WHO’s agenda focuses primarily on *H. pylori* antibiotic resistance as the main cause of eradication failure (Savoldi et al., 2018; Tacconelli et al., 2018). Statistics on *H. pylori* antibiotic resistance papers published each year can help us understand the topic’s development trend. Publications on *H. pylori* antibiotic resistance have generally increased over the past few years. It can be divided into two stages, including a period of rising volatility (2013–2017), and a period of continuous growth (2017–2022). In the period following 2017, this field began to receive extensive attention, based on the increasing number of papers published each year. A possible explanation is that authoritative Consensus guidelines were issued. The publication of “Discovery, research, and development of new antibiotics: the WHO priority list of antibiotic-resistant bacteria and tuberculosis” aroused scientists’ enthusiasm for *H. pylori* antibiotic resistance, which greatly contributed to its development (Tacconelli et al., 2018). According to the polynomial fitting curve, despite a slight fluctuation in published papers in the last 10 years, it can be predicted that the number of published papers will continue to grow positively.

Based on national contributions, China published the most articles worldwide, indicating China has become a major player in *H. pylori* antibiotic resistance research. In addition, there were four Chinese institutions in the top 10 most productive institutions, including Shanghai Jiao Tong University, Nanchang University, Peking University, and Sun Yat-sen University, indicating that Chinese research institutions have become increasingly concerned with *H. pylori* antibiotic resistance. However, in comparison to China, the United States had relatively high Nc and Na, and it ranked first among all countries in terms of both Nc and H-index, indicating a greater influence of academia in this field. Notably, Germany, United Kingdom, and France all exhibit a high Na, suggesting that the caliber of academic works from these countries is widely acknowledged on a global scale. According to these findings, countries should prioritize promoting original innovation and creative discovery in academia, as the number of publications alone cannot fully capture academic influence. Chinese scholars and institutions should make more efforts to improve the quality of their research in the field of *H. pylori* antibiotic resistance. Further, we found that there is an imbalance in the global distribution of current research in this particular field, with papers of high quality and large scale concentrated in developed countries. This may be because governmental expenditure on healthcare is higher in these countries.

Based on the analysis of authors and cited authors, Graham, David Y from Baylor College of Medicine has the highest productivity and citation rates among all authors in the field of *H. pylori* antibiotic resistance, with 44 publications and 2,807 citations. During the period 2013 to 2022, Graham, David Y published every year except 2022. Graham, David Y’s team focused on the efficacy of different therapies for *H. pylori* eradication and the resistance rate of *H. pylori* to different antibiotics. In 2018, David Y Graham’s team conducted a comprehensive assessment of global antibiotic resistance patterns spanning a decade and found that clarithromycin, levofloxacin, and metronidazole resistance exceeded the threshold across most WHO regions (Savoldi et al., 2018). Moreover, Gisbert Javier P, Megraud Francis, Wu Ming-Shiang, and Lu Hong et al. also made great contributions to *H. pylori* antibiotic resistance research.

The assessment of scholarly journals can help authors in selecting the most fitting journals for their research outcomes. Generally, a journal’s impact and its articles’ impact are closely related (Callaham et al., 2002). IF is a metric that can gauge the influence of a journal by measuring its citation rate, academic rigor, and paper quality. Among the top 10 journals with the most papers published, five journals had an IF greater than five, and *International Journal of Molecular Sciences* had the highest IF (6.208). This indicates that the research conducted in this field holds significant scholarly worth and is widely acknowledged. The most productive journals for research on *H. pylori* antibiotic resistance were *Helicobacter*, *World Journal of Gastroenterology*, and *Frontiers in Microbiology*. In addition to producing high volumes, these journals maintain a high citation frequency, which contributes to the dissemination of research findings. Journals with high impact factors and high citation frequencies in this field include *Gastroenterology*, *GUT*, *American Journal of Gastroenterology*, and *Cochrane Database of Systematic Review*. In these journals, scholars can promote their ideas more easily. *H. pylori* antibiotic resistance involve multiple research areas including microbiology, Gastroenterology Hepatology, Pharmacology Pharmacy, Infectious Diseases, Biochemistry Molecular Biology, Medicine General Internal, Immunology, Chemistry Medicinal, Chemistry Multidisciplinary, Biotechnology Applied Microbiology, etc.

### 4.2. Knowledge base

A co-citation analysis can uncover the underlying trends of research literature categories over a specified duration, where studies with a high co-citation ranking are frequently deemed as the foundational research in a particular field (Yang et al., 2019). Below are the top 10 co-cited references related to *H. pylori* antibiotic resistance. Malfertheiner P published “Management of *Helicobacter pylori* infection-the Maastricht IV/Florence Consensus Report (Malfertheiner et al., 2012)” and “Management of *Helicobacter pylori* infection-the Maastricht V/Florence Consensus Report (Malfertheiner et al., 2017)” in *GUT* in 2012 and 2017, which were the most cited papers, with 593 and 506 co-citations. During this study, a group of specialized professionals in the field were assembled to evaluate and deliberate on all pertinent clinical data, with the aim of developing clinical guidelines for the management of *H. pylori*. This research provides a theoretical reference for the clinical management of *H. pylori*. The third co-cited paper, “*Helicobacter pylori* treatment in the era of increasing antibiotic resistance (Graham and Fischbach, 2010),” was published by Graham DY in *GUT* in 2010. This article pointed out that STT has been greatly reduced in effectiveness due to resistance to clarithromycin but it is still used as a first-line treatment. This article recommends that when there is a lack of effective treatment plans, clinicians can try a 14-day concomitant quadruple treatment regimen, a 10-day sequential treatment, or a 14-day bismuth-containing quadruple treatment regimen. The fourth co-cited paper, “*Helicobacter pylori* resistance to antibiotics in Europe and its relationship to antibiotic consumption (Megraud et al., 2013)” was published by Megraud F in *GUT* in 2013. In this study, the researchers evaluated the resistance rate of *H. pylori* in Europe. It was found that among the 2,204 patients incorporated, clarithromycin’s *H. pylori* resistance rate was 17.5%, levofloxacin’s was 14.1%, and metronidazole’s was 34.9%. In addition, it noted that quinolone use correlates with levofloxacin resistance and long-acting macrolides correlate with clarithromycin resistance. The fifth co-cited paper, “ACG clinical guideline: treatment of *Helicobacter pylori* infection (Chey et al., 2017),” was published by Chey WD in *American Journal of Gastroenterology* in 2017. This article is a consensus guide, which is different from expert consensus based on international data. This study focuses on collecting research results for North America. The suggestions provided by this guide will be more suitable for researchers in North America. The sixth co-cited paper, “Prevalence of antibiotic resistance in *Helicobacter pylori*: a systematic review and meta-analysis in World Health Organization regions (Savoldi et al., 2018),” was published by Savoldi A in *Gastroenterology* in 2018. Through systematic evaluation and meta-analysis, this article evaluates the distribution of antibiotic resistance among *H. pylori* in the global population. In this study, the researchers collected 178 studies, including 1,4265 isolates from 66 countries. Results showed that most regions had exceeded 15% of the primary and secondary resistance rates of metronidazole, clarithromycin, and levofloxacin. The seventh co-cited paper, “*H pylori* antibiotic resistance: prevalence, importance, and advances in testing (Mégraud, 2004),” was published by Megraud F in *GUT* in 2004. This is a review article that systematically reviews the resistance rate of *H. pylori* to different antibiotics, the results of the antibiotic drug sensitivity experiments, and the detection technology of the *H. pylori* resistance. The eighth co-cited paper, “Global prevalence of *Helicobacter pylori* infection: systematic review and meta-analysis (Hooi et al., 2017),” was published by Hooi JKY in *Gastroenterology* in 2017. Based on the analysis of 184 samples from 62 countries, this article evaluates the epidemic rate of pylori in the world. The results showed that *H. pylori* prevalence varied widely between regions. The ninth co-cited paper, “The Toronto consensus for the treatment of *Helicobacter pylori* infection in adults (Fallone et al., 2016),” was published by Fallone CA in *Gastroenterology* in 2016. In this consensus, a comprehensive review of the literature production on the management of *H. pylori* infection has been conducted, along with updated recommendations for eradication therapy. The tenth co-cited paper, “Review article: the global emergence of *Helicobacter pylori* antibiotic resistance (Thung et al., 2016),” was published by Thung I in *Alimentary Pharmacology and Therapeutics* in 2016. This article systematically summarizes the latest trend of *H. pylori* antibiotics and current treatment paradigms. Among the top 10 co-cited articles, research reviews provide an overview of the knowledge base in the field, summarize long-term basic research results on *H. pylori* antibiotic resistance, and provide theoretical guidance for researchers in the area of gastrointestinal diseases.

### 4.3. Hotspots and frontiers

We can attain a more profound comprehension of the literature production and the research patterns in a specific discipline of *H. pylori* infection. Among the more representative keywords are: triple therapy, clarithromycin resistance, prevalence, gastric cancer, *in vitro*, *Escherichia coli*, sequential therapy, quadruple therapy, double blind, mutation, gene, antibacterial activity, proton pump inhibitor, rescue therapy, and *Staphylococcus aureus*. As a result of these keywords, we can summarize the general situation of *H. pylori* antibiotic resistance-related fields, including: (1) clarithromycin tolerance strongly reduces the effectiveness of triple therapy (Torres et al., 2001; Álvarez et al., 2009); (2) Chronic gastritis caused by *H. pylori* is closely associated with gastric cancer risk (Parsonnet et al., 1991; Sipponen et al., 1992); (3) As an alternative to first-line empirical therapy, bismuth-containing quadruple therapy has been demonstrated to be effective and safe. In addition, sequential therapy or a non-bismuth quadruple therapy is recommended if bismuth-containing quadruple therapy is unavailable (Malfertheiner et al., 2012); (4) Among *H. pylori* with clarithromycin-resistant strains, mutations of the 23S rRNA gene were associated with failure of the eradication treatment (Chen et al., 2017; Matta et al., 2018); (5) Antimicrobial activity and efficacy are the focus of this field of research (De et al., 2009; Wylie et al., 2022); (6) Rescue therapy, which is the treatment after the failure of *H. pylori* eradication therapy (Gisbert, 2008); (7) Research in this area relies on the prevalence of *H. pylori* in different regions (Thung et al., 2016; Savoldi et al., 2018).

The synthesis of keyword clustering information enables us to precisely delineate the research focus and scope within this field (Kokol et al., 2022). Cluster 1 predominantly comprises keywords associated with *H. pylori* infection research, including pathogenic mechanisms, virulence factors, gastrointestinal disorders, cell experiments, molecular biology, inflammation, immune response, and alternative therapies with natural products. Future research in *H. pylori* infection could focus on identifying novel pathogenic mechanisms and virulence factors that contribute to the development of gastrointestinal disorders. Additionally, studies may explore the role of inflammation and immune responses in *H. pylori* infection, as well as the potential benefits of alternative therapies and natural products to mitigate symptoms and prevent disease progression. Cluster 2 primarily contains keywords linked to treatment approaches and therapeutic effectiveness, such as eradication therapies, antibiotics, drugs, study methods, and guideline reports. Future studies in treatment approaches and therapeutic effectiveness for *H. pylori* infection may involve the development of new eradication therapies and the optimization of existing ones. Scientific research could also focus on the identification of more effective antibiotics and drugs, as well as the establishment of standardized study methods and guidelines to improve clinical outcomes and patient care. Cluster 3 mainly consists of keywords connected to the antibiotic resistance in *H. pylori*, including resistance to various antibiotics, genes, mutations, and detection methods using molecular techniques. With the increasing prevalence of antibiotic resistance in *H. pylori*, future research may concentrate on understanding the underlying genetic mechanisms and mutations that contribute to this resistance. Additionally, the development of more sensitive and accurate detection methods using molecular techniques could help monitor and track the emergence of resistant strains, ultimately informing better treatment strategies. Cluster 4 is chiefly composed of keywords concerning various probiotics and gut microbiota related to *H. pylori*, as well as bowel disease, symptoms, prevention, and treatment. The role of probiotics and gut microbiota in *H. pylori* infection presents a promising area for future research. Studies may investigate the potential benefits of probiotics in preventing and treating *H. pylori*-related bowel diseases and symptoms. Furthermore, scientific research could focus on understanding the complex interactions between *H. pylori* and the gut microbiota, which may lead to new strategies for maintaining a healthy gut environment and preventing infection. Overall, these clusters represent a diverse range of potential research directions, reflecting the multifaceted nature of *H. pylori* antibiotic resistance and the ongoing need for innovative approaches to combat this global health challenge.

According to the chronological presentation of co-cited references and keywords, vonoprazan, 23S rRNA, whole genome sequencing, probiotics, gut microbiota, biofilm, mucosa-associated lymphoid tissue lymphoma, and bismuth have consistently remained as focal points of shared interest over time. Vonoprazan is a novel potassium-competitive acid blocker that suppresses the production of gastric acid by inhibiting H^+^, K^+^ ATPase enzyme in gastric parietal cells and has been used in Japan to eradicate *H. pylori* infection (Sugimoto and Yamaoka, 2018; Yang et al., 2018). According to Min Li et al., vonoprazan has demonstrated greater efficacy than conventional proton pump inhibitor-based therapy in eliminating clarithromycin-resistant *H. pylori* strains (Li et al., 2018). Following the sequencing of multiple *H. pylori* genomes, Schlesinger, N. et al. reported that DNA arrays were a promising method for investigating the genetic diversity of *H. pylori* strains (Salama et al., 2000). In a report by Ge Wang et al., the primary cause of *H. pylori*’s resistance to clarithromycin was attributed to point mutations in the variable area of the 23S rRNA gene. These mutations cause a structural alteration in the clarithromycin binding sites, leading to reduced sensitivity and binding (Wang and Taylor, 1998). In recent times, there have been indications that combining probiotics with standard treatment can enhancing the therapy of *H. pylori* by augmenting eradication rates and mitigating the side effects of current pharmacotherapy (Lü et al., 2016; McFarland et al., 2016; Feng et al., 2017; Wang et al., 2017). However, whether probiotic supplementation contributes to *H. pylori* eradication remains controversial. Yini Dang et al. found that probiotics with triple therapy plus 14 days of treatment did not result in a greater reduction of *H. pylori* infection than placebo (Dang et al., 2014; Lu et al., 2016). The biofilm is a surface-associated community of bacteria embedded in an extracellular polymeric matrix. The biofilm that *H. pylori* forms on gastric mucosa protects it from antimicrobials. Biofilm plays an important role in the failure of antibacterial therapy, so exploring the molecular mechanisms of *H. pylori* biofilm formation has become a research hotbed (Hathroubi et al., 2018, 2020). Gastric mucosa-associated lymphoid tissue lymphoma (MALT) is an indolent B-cell neoplasm strongly associated with *H. pylori* infection. In epidemiological studies, *H. pylori* was found in over 90% of patients with gastric MALT lymphoma. Studies have shown that *H. pylori* is capable of stimulating lymphoma cell growth through mechanisms mediated by T cells (Wotherspoon et al., 1991; Zucca et al., 1998). Several studies have shown that bismuth-containing quadruple therapy is a highly effective treatment for eradicating *H. pylori*, including resistant strains as well as susceptible strains (Laine et al., 2003; Malfertheiner et al., 2011; Dore et al., 2016). These studies have provided insight into future developments of *H. pylori* antibiotic resistance.

## 5. Conclusion

This research utilized bibliometrics and visualization technology to succinctly outline and evaluate the worldwide state of research, development trends, prominent research avenues, and emerging focal points concerning antibiotic resistance in *H. pylori*. Our bibliometric study shows that the quantity of articles on *H. pylori* antibiotic resistance has experienced fluctuations over the past decade, although a general trend of steady growth remains unmistakable, indicating that antibiotic resistance in *H. pylori* is an important or urgent research topic. We have identified the primary contributors, publications, and regions within the research domain. China published the largest number of documents, while the US had the greatest influence. *H. pylori*’s drug resistance mechanism is a current research focus. There has been considerable interest in *H. pylori*’s antibiotic resistance genes. The role of RdxA in metronidazole sensitivity and the mutation of 23S rRNA gene have become potential hotspots in *H. pylori* resistance research. Additionally, *H. pylori* biofilm formation and its potential role in pathogenesis have received considerable attention. In the eradication treatment of *H. pylori*, vonoprazan and chain fatty acids have also been considered hot topics.

The bibliometric study on *H. pylori* antibiotic resistance provides valuable insights for researchers in this field. For guideline makers, this research provides a comprehensive overview of the current state of knowledge, enabling them to make informed decisions when formulating evidence-based policies and recommendations. Novice researchers can benefit from the study by gaining a clear understanding of the research landscape, hot spots, and trends, which can guide their future investigations and help them identify potential areas of focus. Finally, for clinicians, our research offers a valuable resource to stay updated on the latest findings and advancements in the field, ultimately allowing them to provide better care for their patients suffering from *H. pylori* infections and antibiotic resistant strains.

## 6. Strengths and limitations

We used VOSviewer, CiteSpace, and R (version 4.2.2) simultaneously to perform a comprehensive bibliometric analysis of the research on *H. pylori* antibiotic resistance. Multiple statistical tools make our statistical results more objective and accurate. This research offered a more comprehensive understanding of changing research priorities and patterns than conventional reviews. Nevertheless, it is not without its limitations. First, this study only included the WOS core database; other databases, such as PubMed, Medline, and Google Scholar, were not considered. As a result, the included literature production may be incomplete. Second, our research was restricted to reviews and articles published only in the English language, which may have led to the exclusion of certain high-quality papers. Third, certain inconsistencies may exist. For instance, an author may be affiliated with different institutions at different points in time. Finally, the selected publications were released between 2013 and 2022, and some studies published in 2023 with a low Nc were not considered. Therefore, this research has some latency.

## Data availability statement

The raw data supporting the conclusions of this article will be made available by the authors, without undue reservation.

## Author contributions

HS and MZ proposed and designed the entire study and revised and edited the final version. CYuan extensively reviewed the relevant literature production, established the inclusion and exclusion criteria for the study, and drafted the manuscript. CYu and SZ screened the literature production while QS identified the controversial portion. MX extracted and compiled data from the documents analyzed in this study. CYuan and CYu used softwares to visualize the data. All authors contributed to the article and approved the submitted version.

## Funding

This study was funded by grants from the National Natural Science Foundation of China (nos. 81703920 and 82004427), China Postdoctoral Science Foundation (no. 2019M662790), Science and Technology Innovation Program of Hunan Province (no. 2021RC3101), Guidance Project of Academician Liu Liang’s Expert Workstation (no. 22YS003), Key Project of Science Research of Hunan Provincial Education Department (no. 21A0240), and Open-competing Disciple Construction Project of Hunan University of Chinese Medicine (no. 22JBZ027).

## Conflict of interest

The authors declare that the research was conducted in the absence of any commercial or financial relationships that could be construed as a potential conflict of interest.

## Publisher’s note

All claims expressed in this article are solely those of the authors and do not necessarily represent those of their affiliated organizations, or those of the publisher, the editors and the reviewers. Any product that may be evaluated in this article, or claim that may be made by its manufacturer, is not guaranteed or endorsed by the publisher.

## Footnotes

^1^ http://www.bioinformatics.com.cn/

## Abbreviations

AMPCs: Proton pump inhibitor clarithromycin and amoxicillin
CAMs: Clarithromycins
*H. pylori*: *Helicobacter pylori*
IF: The impact factor
JCR: The journal citation report
Nc: The number of citations
Np: The number of publications
Na: The number of average citations
PPI: Proton pump inhibitors
SCI-expanded: Science citation index expanded
STT: Standard triple therapy
WoSCC: Web of science core collection
