# Supplementary material for: Research on antibiotic resistance in Helicobacter pylori: a bibliometric analysis of the past decade
Source: Front Microbiol. 2023 Jun 14;14:1208157. doi: 10.3389/fmicb.2023.1208157 (PMC10301835; doi:10.3389/fmicb.2023.1208157)
Supplement: Supplementary file 1 [file Table_1.DOCX]

**Supplementary Table 1.** Results of the synthetic knowledge synthesis.

| Theme | Colour | More frequent keywords | Categories |
| --- | --- | --- | --- |
| *H. pylori* infection | red | *in vitro* (255), identification (115), gastric cancer (110), gastritis (108), mechanisms (88), CagA (84), inflammation (77), pathogenesis (70), nanoparticles (65), biofilm formation (61), VacA (53), virulence factors (52), crystal structure (51), ulcer (44), NF-kappa B (43), gene expression (35), apoptosis (33), adhesion (29), chitosan (28), flavonoids (25), vacuolating cytotoxin (21) | Pathogenic mechanisms and virulence factors, gastrointestinal disorders, cell experiments and molecular biology, inflammation and the immune response, alternative therapies and natural products |
| Treatment strategies and therapy efficacy | green | eradication (667), clarithromycin (538), triple therapy (485), metronidazole (337), amoxicillin (291), meta-analysis (234), sequential therapy (195), quadruple therapy (173), levofloxacin (163), bismuth (132), rescue therapy (105), tetracycline (102), omeprazole (89), proton pump inhibitor (82), consensus report (78), concomitant therapy (76), vonoprazan (60), randomized trial (48) | Eradication therapies, antibiotics & drugs, study methods and guideline report |
| Antibiotic resistance | blue | prevalence (285), clarithromycin resistance (187), mutations (118), antimicrobial susceptibility (109), metronidazole resistance (88), gene (81), 23S ribosomal RNA (66), PCR (56), point mutations (46), fluoroquinolone resistance (43), 13C-urea breath test (33), polymerase chain reaction (33), amoxicillin resistance (32), genotypes (30), stool antigen test (26) | Resistance to different antibiotics, genes and mutations, detection methods and molecular techniques |
| Probiotics and gut microbiota | yellow | probiotics (147), gut microbiota (100), prevention (52), *saccharomyces boulardii* (52), antibiotic associated diarrhea (49), irritable bowel syndrome (39), lactic acid bacteria (38), inflammatory bowel disease (35), malt lymphoma (30), chain fatty acids (29), dysbiosis (26) | Probiotics and microbiota, bowel disease and symptoms, prevention and treatment |

The figures within brackets indicate the quantity of papers where the keyword was present.
